# Biallelic Variants in Seven Different Genes Associated with Clinically Suspected Bardet–Biedl Syndrome

**DOI:** 10.3390/genes14051113

**Published:** 2023-05-19

**Authors:** Hamed Nawaz, Mujahid, Sher Alam Khan, Farhana Bibi, Ahmed Waqas, Abdul Bari, Fardous, Niamatullah Khan, Nazif Muhammad, Amjad Khan, Sohail Aziz Paracha, Qamre Alam, Mohammad Azhar Kamal, Misbahuddin M. Rafeeq, Noor Muhammad, Fayaz Ul Haq, Shazia Khan, Arif Mahmood, Saadullah Khan, Muhammad Umair

**Affiliations:** 1 Department of Biotechnology and Genetic Engineering, Kohat University of Science & Technology (KUST), Kohat 26000, Pakistan; hamedwazir@gmail.com (H.N.); sakmarwat79@gmail.com (S.A.K.); abdulbari000999@gmail.com (A.B.); niamatexclusive@gmail.com (N.K.); afaqi.k21@gmail.com (N.M.); dr.noor@kust.edu.pk (N.M.); 2 Center of Animal Nutrition, Directorate General of Livestock & Dairy Development, Peshawar 25000, Pakistan; mkmqau@gmail.com; 3 Department Zoology, Division of Science and Technology, University of Education, Lahore 54782, Pakistan; ravian643@gmail.com; 4 Department of Medical Lab Technology (MLT), Kohat University of Science & Technology (KUST), Kohat 26000, Pakistan; fardousjamal26@gmail.com; 5 Faculty of Science, Department of Biological Sciences (Zoology), University of Lakki Marwat, Lakki Marwat 28420, Pakistan; amjadkhanqau123@hotmail.com; 6 Department of Anatomy, KMU Institute of Medical Sciences (KIMS), Kohat 26000, Pakistan; drsohailparacha@gmail.com; 7 Molecular Genomics and Precision Medicine, ExpressMed Laboratories, Block Zinj, Manama 359, Bahrain; qamar.alam1@gmail.com; 8 Department of Pharmaceutics, College of Pharmacy, Prince Sattam Bin Abdulaziz University, Al-kharj 11942, Saudi Arabia; ma.kamal@psau.edu.sa; 9 Department of Pharmacology, Faculty of Medicine, Rabigh, King Abdulaziz University, Jeddah 21589, Saudi Arabia; marafeeq@kau.edu.sa; 10 Department of Radiological Sciences, College of Applied Medical Sciences, King Saud Bin Abdulaziz University for Health Sciences, Riyadh 12271, Saudi Arabia; haqf@ksau-hs.edu.sa; 11 Hafeez Institute of Medical Sciences, Islamabad 44000, Pakistan; shazia.phdbt20@iiu.edu.pk; 12 Department of Biological Sciences, International Islamic University Islamabad, H-10, Islamabad 44000, Pakistan; 13 Center for Medical Genetics, Hunan Key Laboratory of Medical Genetics, School of Life Sciences, Central South University, Changsha 410078, China; khanbiochemist007@gmail.com; 14 Medical Genomics Research Department, King Abdullah International Medical Research Center (KAIMRC), King Saud Bin Abdulaziz University for Health Sciences, Ministry of National Guard Health Affairs (MNGH), Riyadh 12271, Saudi Arabia; 15 Department of Life Sciences, School of Science, University of Management and Technology (UMT), Lahore 14611, Pakistan

**Keywords:** ciliopathy, Bardet–Biedl syndrome, *IFT27*, *BBIP1*, *WDPCP*, *LZTFL1*, *MKKS*, *BBS1*, *BBS5*, polydactyly, obesity, intellectual disability

## Abstract

Bardet–Biedl syndrome (BBS) is a rare clinically and genetically heterogeneous autosomal recessive multi-systemic disorder with 22 known genes. The primary clinical and diagnostic features include six different hallmarks, such as rod–cone dystrophy, learning difficulties, renal abnormalities, male hypogonadism, post-axial polydactyly, and obesity. Here, we report nine consanguineous families and a non-consanguineous family with several affected individuals presenting typical clinical features of BBS. In the present study, 10 BBS Pakistani families were subjected to whole exome sequencing (WES), which revealed novel/recurrent gene variants, including a homozygous nonsense mutation (c.94C>T; p.Gln32Ter) in the *IFT27* (NM_006860.5) gene in family A, a homozygous nonsense mutation (c.160A>T; p.Lys54Ter) in the *BBIP1* (NM_001195306.1) gene in family B, a homozygous nonsense variant (c.720C>A; p.Cys240Ter) in the *WDPCP* (NM_015910.7) in family C, a homozygous nonsense variant (c.505A>T; p.Lys169Ter) in the *LZTFL1* (NM_020347.4) in family D, pathogenic homozygous 1 bp deletion (c.775delA; p.Thr259Leufs*21) in the *MKKS*/*BBS5* (NM_170784.3) gene in family E, a pathogenic homozygous missense variant (c.1339G>A; p.Ala447Thr) in *BBS1* (NM_024649.4) in families F and G, a pathogenic homozygous donor splice site variant (c.951+1G>A; p?) in *BBS1* (NM_024649.4) in family H, a pathogenic bi-allelic nonsense variant in *MKKS* (NM_170784.3) (c.119C>G; p.Ser40*) in family I, and homozygous pathogenic frameshift variants (c.196delA; p.Arg66Glufs*12) in *BBS5* (NM_152384.3) in family J. Our findings extend the mutation and phenotypic spectrum of four different types of ciliopathies causing BBS and also support the importance of these genes in the development of multi-systemic human genetic disorders.

## 1. Introduction

Ciliopathy is a heterogeneous, multisystem genetic disorder caused by dysfunction of cilia. The cilia are hair-like outgrowths originating from the centriole and are of two types, motile and non-motile/primary. A typical cilium consists of mainly two parts, the basal body and the axoneme. Between them, a transitional zone/cilial domain is present, which acts like an enter and exit point for cilium and contains transitional fibers/alar sheets [1,2]. The basal body arises from the centriole providing an anchoring site for cilium, while the axoneme is composed of microtubules (the backbone of cilium) surrounded by a special membrane. In the axoneme of motile cilia, the two central doublets are surrounded by 09 pairs of outer doublets, making a (9 + 2) arrangement. In the case of non-motile/primary cilia, the central doublets are absent, making a (9 + 0) arrangement [2,3]. The intra-flagellar transport (IFT) mechanism is essential for trafficking the ciliary protein cargo for building, reabsorbing, and maintaining the newly synthesized cilium in the cell. In the cilium, the IFT cargo vesicle has anterograde and retrograde movement composed of different molecules such as IFT-B and kinesin particles, which are required for anterograde, while IFT-A and dynein are required for retrograde movement [3].

Regardless of the similarities, their function and effect are dramatically different from each other. Primary cilia function like antennae, sensing both mechanical and chemical changes in the microenvironment of the cell, through which they regulate fundamental biological processes, such as sensing extracellular cues and cell signaling (Wnt, Hedgehog, PDGF, etc.), leading to the transcription of target genes and playing a role in tissues homeostasis [2,4]. As already discussed, cilial function is notably associated with the BBS protein’s stability and functionality. Any anomalies in primary cilial/BBS proteins will lead to diverse clinical manifestations, ranging from a single organ to multi-systemic disorders, termed as ciliopathy (Bardet–Biedl syndrome, Joubert Syndrome, polycystic kidney disease, etc.), while dysfunction of motile cilia usually causes disorder of situs-inversus, hydrocephalus, respiratory, infertility, etc. [5,6].

Bardet–Biedl syndrome (BBS; MIM, PS209900) is a rare genetic, pleiotropic, multi-symptomatic autosomal recessive ciliopathy disorder. It is characterized by a wide spectrum of clinical features that affect different body systems, including polydactyly (hexadactyly), obesity (truncal), retinal degeneration (eye), renal dysfunction (kidney), genital anomalies (hypogonadism), and congenital impairment (learning disabilities). Minor or secondary features are also observed, though not always present in all BBS patients, including diabetes mellitus, liver disease, congenital heart defects, hearing loss, dental anomalies, facial dysmorphism, developmental delay, and many others [7]. Owing to the heterogeneous nature of BBS, clinical diagnosis is performed based on at least four primary features or three primary and two secondary features [8].

Two types of populations, geographically isolated and consanguineous families, have a high rate of BBS incidence. BBS is an autosomal recessive disorder; however, the occurrence of multi-gene involvement is common such as di-genic and tri-genic inheritance. BBS incidence is generally 1:25,000, while it is variable across different ethnicities, approximately 1:17,000 (Kowait), 1:156,000 (Tunisia), 1:65,000 (Arab population), and 1:37,000 (Foreislands) [3,7,9].

Defects in ciliogenesis (cilium formation) result in genetically heterogeneous multi-systematic disorders known as ciliopathies. As almost every cell contains cilia, any defects at the molecular level in the ciliary proteins archetypally affect different organ systems. Cilia play a vital role in signal transduction and enable cell-to-cell/cell-to-surrounding communications [10].

BBS is a multi-systematic ciliopathy disorder characterized by mixed rod–cone dystrophy, with 40% of patients having renal and hypogonadism, 50% of patients suffering from intellectual disability, 2/3 patients having PAP, syndactyly, clinodactyly, or/and brachydactylic, and 70% of the patients experiencing truncal obesity associated with type 2 diabetes, hypertension, and dyslipidemia [3].

To date, genetic analysis has identified 22 genes implicated in causing BBS, among them *BBS1*, *BBS2*, *BBS4*, *BBS5*, *BBS7*, *BBS8* (*TTC8*), *BBS9* (*PTHB1*), *BBS17* (*LZTFL1*), and *BBS18* (*BBIP1*) are responsible for encoding the BBSome protein, while *BSS13* (*MKS1*), *BBS14* (*CEP290*; *TMEM67*), *BBS15* (*WDPCP*), and *BBS16* (*SDCCAG8*) produce the basal body interacting protein, *BBS6* (*MKKS*), *BBS10*, and *BBS12* form the chaperonin complex protein, *BBS11* (*TRIM32*) encodes E3 ubiquitin ligase, *BBS3* (*ARL6*), *BBS19* (*IFT27*), and *BBS20* (*IFT172*) give rise to a GTPase protein complex, *BBS21* (*C8ORF37*) plays an important role in primary cilia function and is involved in the development of BBS, and *BBS22* (*CEP19*) is responsible for encoding centrosomal and ciliary proteins [6,7,11].

Herein, we characterize 10 families with hallmark features of BBS. Genetic and molecular analysis revealed four novel and six already reported mutations in seven different genes previously associated with BBS.

## 2. Materials and Methods

### 2.1. Ethical Approval

The families presented here were ascertained from remote, poorly developed areas of Pakistan. Written informed consent for the publication of data and photographs were obtained from the parents (father) in compliance with the Helsinki Declaration. The IRB of UMT, Lahore [Dated: 3 January 2022; Ref# DLSBBC-2022-04], Pakistan and ethical research committee of the Kohat University of Science and Technology, Kohat, [dated: 16 July 2021; Certificate number: 1988] Pakistan, approved the research study.

### 2.2. DNA Isolation

**Figure 1:**
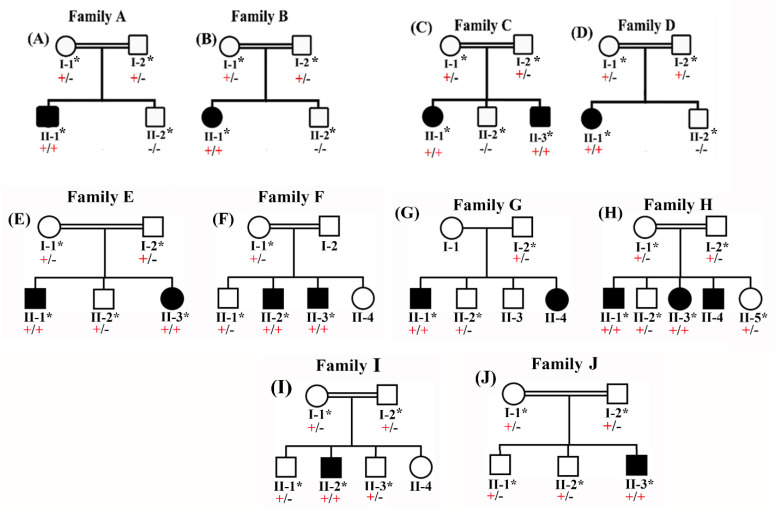
(**A**–**J**) Pedigree of families showing autosomal recessive BBS.

Ten BBS families were examined for the primary and secondary features, pedigrees were constructed, and blood was collected in EDTA tubes from all the members labeled with asterisks in the pedigrees (Figure 1A–H). Patients’ histories highlighted different prenatal, postnatal, and neonatal problems. Subsequently, DNA extraction and quantification were performed using standard methods [12].

### 2.3. (SNP)-Microarrays

(SNP)-microarrays were performed on families I and F using standard methods [13] to rule out uniparental disomy (UPD).

### 2.4. Whole-Exome Sequencing (WES)

In the present study, genomic DNA from at least one affected individual from each family was subjected to WES. For each sample, 4 ug of genomic DNA was enriched for the target region of all humans with consensus coding sequence (CCDS) exon 14 with Agilent’s SureSelect Human All Exon Kit V2 and afterward sequenced on an Illumina Genome Analyzer II with 100 bp single end reads. Later on, all the reads were aligned with hg19 (GRCh37) human assembly via a Burrows–Wheeler Aligner v 0.7.5. The variants were called using different programs, including PINDEL v 0.2.4 t, SAM tools v 0.1.18, and Exome Depth v 1.0.0. BaseSpace was used for analyzing the obtained variants. Based on the pedigrees with autosomal recessive inheritance, we were interested in rare disease-causing homozygous and/or compound heterozygous variants. WES filtration steps include inheritance patterns, OMIM gene list of BBS genes, biallelic variants, compound heterozygous variants, and minor allele frequency (MAF) >0.001 in gnomAD and EXAC, as described previously [14,15].

### 2.5. Sanger Sequencing

All the exons, introns, and upstream and downstream UTRs were retrieved from the Ensemble genome browser, and primers were designed using primer-3-software. The identified variants were Sanger sequenced in all the affected and unaffected individuals according to standard methods [16,17].

### 2.6. In Silico Analysis

Functional effect for variants was checked using online available bioinformatics tools such as SIFT, MutationTaster, CADD, and VarSome. The frequency of the identified mutation in the general population was determined using ExAC, gnomAD, and 165 in-house exomes. Conservation of the mutated amino acids was checked using NCBI-HomoloGene [18].

## 3. Results

### 3.1. Clinical Evaluation

Clinical features of BBS patients in the families are also described in Table 1.

### 3.2. Family A (IFT27)

**Figure 2:**
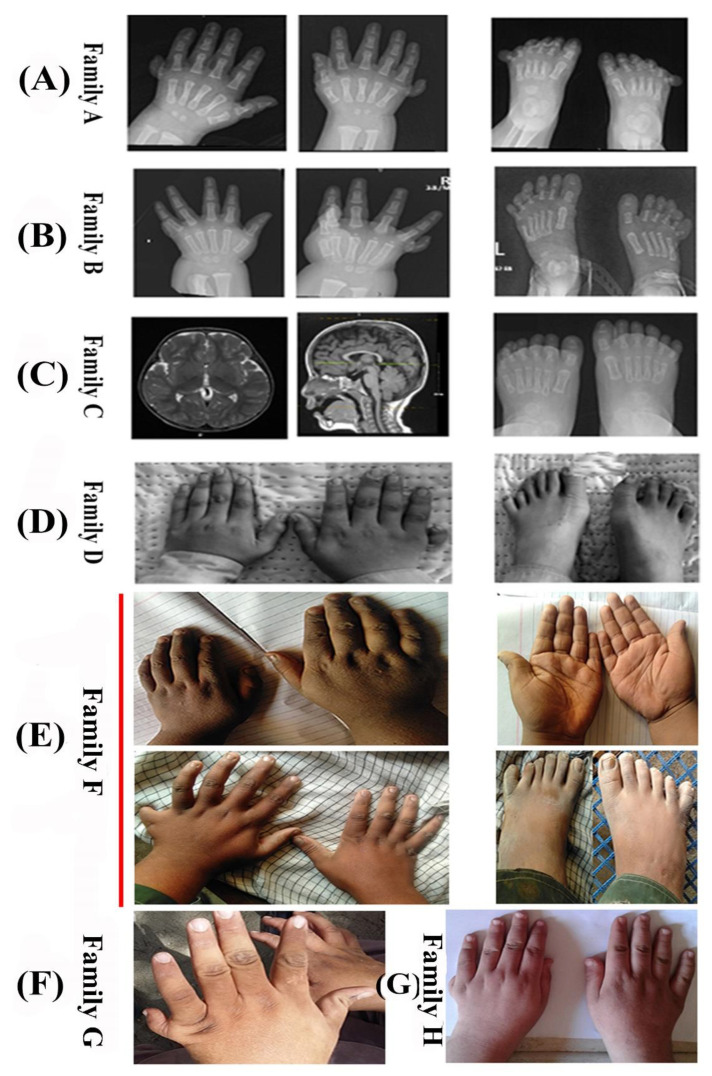
(**A**–**G**) Radiographs and pictures of hands and feet of the affected individuals in families (**A**–**D**) and (**F**–**H**).

Family A showed a consanguineous pedigree. The affected individual (boy; II-I) of family A was 12 years old, and his parents were first cousins. The proband shared typical phenotypes of BBS, including bilateral post-axial polydactyly (both hands and in feet) with hypoplastic phalanges and accessory fingers, obesity, kidneys were normal with left grade II hydronephrosis and proximal left ureteric dilation measure of 0.3 cm, mild learning disability, speech difficulties, mild hearing impairment, and retinitis pigmentosa (Figure 2A). At the age of 4, the proband (II-I) experienced weakening vision and was referred to the ophthalmic department. He is non-aggressive, happy, and did not feel pain when blood was drawn from him.

### 3.3. Family B (BBIP1)

The affected individual (II-I) was a 14-year-old girl and a product of first-degree consanguineous marriage. The phenotypes observed in the proband (II-1) included ID, obesity, mantel retardation, renal abnormalities (bilaterally enlarged echogenic kidneys), vision impairment, and bilateral polydactyly. Furthermore, the affected individual also has an abnormal-shaped spleen with a lobulated outline in addition to a smaller adjacent spleen (polysplenia) and poor appreciation of the coronal suture in the skull (Figure 2B).

### 3.4. Family C (WDPCP)

The affected individuals (II-1-girl, II-3-boy) were 8 and 11 years old, and their parents were first-degree cousins. Both the affected individuals (II-1, II-3) shared typical phenotypes of BBS, including obesity, bilateral post-axial polydactyly with a complete left separate extra toe, renal anomalies (bilateral grade II hydronephrosis), mild learning disability, speech difficulties, mild hearing impairment, and vision anomalies (Figure 2C). In addition, the affected individual (II-3) had hypogonadism with a micropenis (images not shown). Both the affected individuals had removed the extra digit in their hands with the help of a surgical procedure.

### 3.5. Family D (LZTFL1)

The affected individual (II-1) was a 15-year-old girl born to a consanguineous couple. The affected individual revealed obesity, mild learning disability, speech difficulties, mild hearing impairment, kidney stones, and retinitis pigmentosa (RP). The affected individual (II-1) had unilateral post-axial polydactyly in the right hand and unilateral post-axial polydactyly in the left foot (Figure 2D). At the age of 10 years, the proband (II-1) experienced vision abnormalities and became legally blind at the age of 14 years. She also had a problem with appetite and developed obesity. Renal function tests and metabolic screening showed a normal range of different parameters. The CBC (complete blood count) and heart rate were normal. There was no previous family history of such a disorder, and the parents were normal.

### 3.6. Family E (MKKS/BBS6)

Family E has two affected individuals (II-1, II-3) from a consanguineous marriage. Both the parents and a normal individual also participated in this study. The typical features of BBS patients in this family include post-axial polydactyly, intellectual disability, obesity, retinitis pigmentosa (RP), and hypogonadism. They also presented some secondary features, including renal complications, blindness, developmental delay, diabetes mellitus, and speech and language difficulties.

### 3.7. Family F (BBS1)

In family E, the affected male individuals (II-2, II-3) were born into a consanguineous marriage. Primary features of BBS were presented by both patients, including truncal obesity, polydactyly, intellectual disability, hypogonadism, and retinitis pigmentosa (RP). Patient (II-2) had post-axial polydactyly only in his feet, but patient (III-3) showed unilateral post-axial polydactyly in his hands but bilateral polydactyly in his feet (Figure 2E). Moreover, they also showed mild features of neurological speech impairment and learning difficulties, poor coordination, developmental delay, nystagmus, strabismus, renal insufficiency, and hypodontia.

### 3.8. Family G (BBS1)

Family F showed a non-consanguineous pedigree with two affected individuals (II-1, II-4), but only patient II-1 was available at the time of blood collection. He presented all typical features of BBS, including unilateral left-hand post-axial polydactyly, intellectual disability, visual impairment, hypogonadism, and obesity (Figure 2F). In addition to these, he also showed some secondary features, including a micropenis, decreased testicular size, developmental delay, retinal degeneration, and speech and language deficiencies. 

### 3.9. Family H (BBS1)

Family F presented a consanguineous pedigree and had three BBS patients, two brothers (II-1, II-4) and a sister (II-3), but only two patients (II-1, II-3) participated in this study. A total of six individuals participated in this study, including parents (II-1, II-2) and their normal offsprings (II-2, II-5), in addition to their two affected children (II-1, II-3). The BBS patients showed typical features of the syndrome, including truncal obesity, intellectual disability, hypogonadism, retinitis pigmentosa, and post-axial polydactyly. Patient II-1 has only unilateral left-hand post-axial polydactyly (Figure 2G), while patient II-3 has bilateral foot post-axial polydactyly. Moreover, they also showed some secondary features of BBS, including developmental delay, poor cognitive and speech ability, cataracts, and renal abnormalities. 

### 3.10. Family I (MKKS)

Family I presented a consanguineous pedigree, exhibiting classic BBS characteristics, which was found in a remote Pakistani village area in the Sindh province of Pakistan. Inter-familial unions are frequent in this area, and residents adhere strictly to its culture and traditions. Pedigree research proved that the condition is inherited autosomal recessively. The affected person, II-2, was 16 years old. The affected person in the current study had several characteristics that are typical of BBS, including obesity, a little intellectual handicap, post-axial polydactyly (PAP) in at least one hand or foot, hypogonadism, and impaired vision that is likely the result of retinal degeneration. The affected person II-2 only had PAP in their right hand, was unable to speak or understand language, and had learning issues (Figure 1I).

### 3.11. Family J (MKKS)

Family J showed a consanguineous pedigree and was recruited from the Khyber Pakhtunkhwa (KP) province of Pakistan, presenting the hallmark features of BBS. Autosomal recessive inheritance was verified by pedigree analysis.

The affected individual (II-3) was 18 years old at the time of genetic and clinical analysis. He showed moderate DD, ID, visual impairment, truncal obesity, ataxia, PAP (surgically removed), and renal issues. He was unable to understand language, had learning difficulties, and was not able to speak. Additional abnormalities such as renal anomalies, diabetes mellitus, short stature, congenital heart malformations, dental anomalies, and thyroid deficiency were not observed (Figure 1J).

### 3.12. Molecular Investigation

(SNP)-microarrays did not reveal any abnormality. WES was performed as described earlier [14,15]. Screening and filtering different homozygous and compound heterozygous variants revealed four novel nonsense mutations in four BBS families that include a homozygous variant (c.94C>T; p.Gln32Ter) in the *IFT27* gene (NM_006860.5) in family A located on chromosome 22q12.3, a homozygous variant (c.160A>T; p.Lys54Ter) in the *BBIP1* gene (NM_001195306.1) in family B located on chromosome 10q25.2, a homozygous variant (c.720C>A; p.Cys240Ter) in the *WDPCP* (NM_015910.7) gene in family C located on chromosome 2p15, and a homozygous variant (c.505A>T; p.Lys169Ter) in the *LZTFL1* (NM_020347.4) gene in family D located on chromosome 3p21.31. The *IFT27* variant was classified as pathogenic, and the *BBIP1* variant was classified as a variant of uncertain significance (VUS), while *WDPCP* and *LZTFL1* variants were classified as likely pathogenic according to ACMG/AMP guidelines [19] (Table 2).

The *IFT27* gene product consists of 185 amino acids. As a result of the transition of C into T at position 94 (c.94C>T), a stop codon is generated at amino acid position 32 (p.Gln32Ter) in family A. Instead of a full-length protein (185 amino acids), a truncated protein product, consisting of only 31 amino acids, is produced, which will be deactivated by nonsense-mediated mRNA decay (NMD), which will eliminate this mRNA-containing premature translation–termination codon (PTC) and no protein product will form at all. 

The *BBIP1* gene encodes a wild-type protein of 92 amino acids in length. A at position 160 is transversed by T (c.160A>T), and as a result, a premature termination codon (PTC) is generated at position 54 in place of lysine (p.Lys54Ter) in family B. So, a mutated truncated protein consisting only of 53 amino acids is produced, which will be immediately degraded by activating the NMD machinery, and no protein product will be produced at all. 

The *WDPCP* gene encodes a protein that is 746 amino acids long. After replacing C with A at nucleotide position 720 (c.720C>A), cysteine in the wild-type protein at amino acid position 240 is substituted by a premature termination codon (p.Cys240Ter) in the mutated protein in family C. Here, instead of a full-length protein (746 amino acids), a truncated protein consisting only of 239 amino acids is produced. The NMD mechanism will be activated, and the mRNA containing this termination codon will be degraded, producing no functional protein. 

The wild-type *LZTFL1* gene encodes a protein of 299 amino acids in length. When A at nucleotide position 505 is substituted by T (c.505A>T), the amino acid lysine at amino acid position 169 is replaced by a premature termination codon (p.Lys169Ter) in family D. As a result of this substitution, a truncated protein of 168 amino acids is produced. In this case, the NMD machinery will also be activated, and the mRNA containing the PTC will be degraded.

Similarly, we also identified a homozygous 1 bp deletion (c.775del; p.Thr259Leufs*21) in the *MKKS* (NM_170784.3) gene in family E, and two already reported *BBS1* variants in an additional three families (F-H). The *MKKS* variant in family E was reported with an extremely low frequency from gnomAD v2.1.1 and was also reported as pathogenic for retinal dystrophy (ClinVar ID: VCV000866319; rs759131391) without any evidence from an independent evaluation at the laboratory level. This frameshift variant was classified as pathogenic (Table 2).

The *MKKS* gene encodes a protein of 570 amino acids in length. In this case, 1 bp deletion (c.775delA) takes place at nucleotide position 775. As a result, the frame shifts and threonine (Thr) at amino acid position 259 is replaced by leucine (Leu) and a premature termination codon (PTC) is generated downstream after 20 amino acids at the 21^st^ position from the point of amino acid substitution (p.Thr259Leufs*21) in family E. As a result, a truncated protein consisting only of 278 amino acids will be produced. This single bp deletion generates a frameshift variant, which is expected to cause a loss of normal protein function via nonsense-mediated mRNA decay (NMD).

Both families F and G revealed the same pathogenic homozygous missense variant c.1339G>A; this was p.Ala447Thr in the *BBS1* (NM_024649.4) gene with an extremely low allele frequency of 0.00010 in the large population cohorts (gnomAD; http://gnomad.broadinstitute.org/, accessed on 2 January 2022). The homozygous variant c.1339G>A in *BBS1* changes an amino acid Ala to Thr at codon 447 in exon-13. Multiple lines of computational evidence predict this variant as probably damaging to the protein structure, function, or protein–protein interaction. This variant was classified as pathogenic according to the recommendation of ACMG/AMP guidelines [19] (Table 2). In both families, guanine (G) was replaced by adenine (A) at nucleotide position 1339 in the *BBS1* gene. As a result, alanine (Ala) at amino acid position 447 was substituted by threonine (Thr). Here, it is necessary to mention that alanine is non-polar, neutral, and hydrophobic, while threonine is polar, neutral, and hydrophilic. So, substituting alanine with threonine will lead to distortion in the protein structure.

In family H, a homozygous donor splice site variant (c.951+1G>A; p?) was identified in the *BBS1* gene (NM_024649.4). This splice site variant was classified as pathogenic based on the ACMG recommendations for the interpretation of sequence variants [19]. It is absent from gnomAD v2.1.1. Substitution at the splicing junction produces an abnormal splicing effect, which is expected to cause a loss of normal protein function via nonsense-mediated mRNA decay or skipping of exon-10

**Figure 3:**
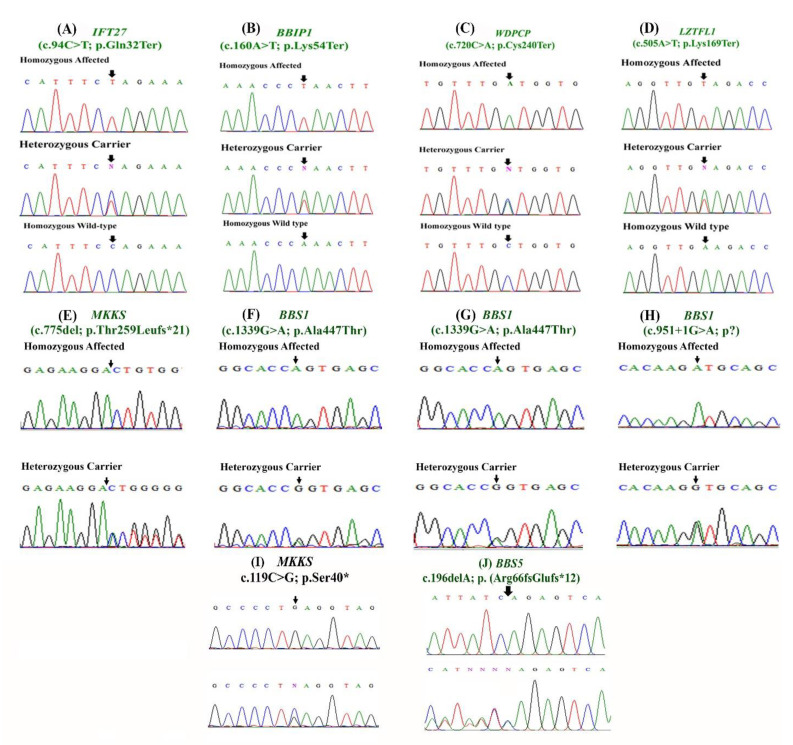
Sanger segregation of the variants identified in families (**A**–**J**). Black arrows represent the position of the identified variants.

In family I, a homozygous nonsense variant (c.119C>G; p.Ser40*) was revealed in the *MKKS* gene located on chromosome 20p12.2 (Figure 3I). The identified variants perfectly segregated with the disease phenotype within the families. This nonsense variant was pathogenic according to ACMG/AMP (Table 2). Here, C at nucleotide position 119 was substituted by G (c.119C>G) in the *MKKS* gene. This nucleotide transversion results in the replacement of serine (Ser) at amino acid position 40 by a premature termination codon. As a result, a truncated protein (39 amino acids) is generated instead of a full-sized protein. Hence, the NMD machinery will be activated and will degrade the truncated protein. 

Similarly, in family J, WES revealed an already reported pathogenic frameshift variant (c.196delA; p.Arg66Glufs*12) in exon 2 of the *BBS5* gene (NM_152384.2). The variant was classified as likely pathogenic according to ACMG/AMP (Table 2). The variant segregated perfectly in the family. *BBS5* encodes a protein of 341 amino acids in length. Here, 1 bp deletion (c.196delA) takes place at nucleotide position 196. As a result, the frame shifts and arginine (Arg) at amino acid position 66 is replaced by glutamic acid (Glu) and a premature stop codon is generated downstream after 11 amino acids at the 12th position from the point of amino acid substitution (66) and a truncated protein consisting only of 76 amino acids is produced. The NMD mechanism will be activated, and the mRNA containing premature termination codon will be degraded.

Both the variants in families I and J have been previously reported to cause BBS phenotypes in the literature. The variant was screened in ExAC, gnomAD, and 145 control exomes to rule out the occurrence of polymorphism and incidence of the identified variants in the general population. The pathogenicity tools indicated that all the variants identified were disease-causing and responsible for the disease phenotype in the patients.

Sanger sequencing of the identified novel and already reported variants were performed using standard methods, and all the variants were perfectly segregated with the disease phenotype within the families (Figure 3A–H). The mutated amino acids in each family were also conserved across different species.

## 4. Discussion

In the present study, we identified four novel homozygous and six previously reported homozygous variants in 10 families exhibiting hallmark features of BBS. The clinical features observed in all the families overlapped with the features reported previously [5,6], such as polydactyly, obesity, hypogonadism, learning/speech disability, slight mental retardation, and retinitis pigmentosa [5,6,20].

**Figure 4:**
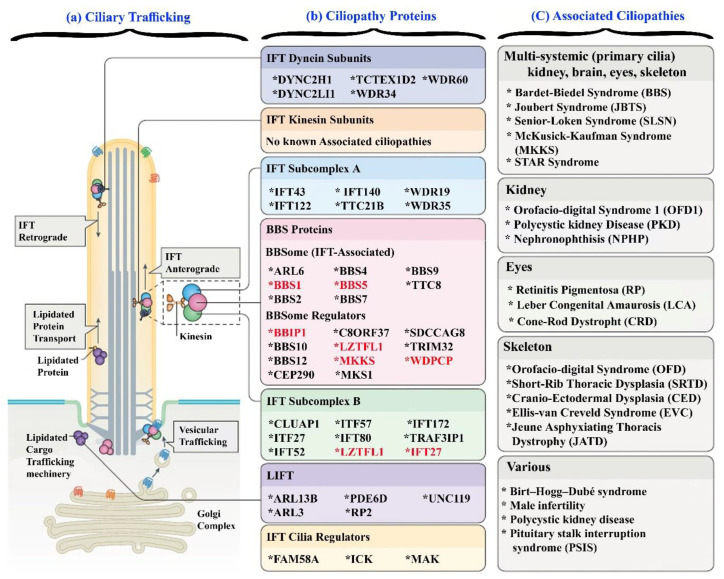
Genes associated with BBS and their function in cilia trafficking. The variants identified in genes investigated in the present study are represented in red.

In this study, we identified a novel variant (c.94C>T; p.Gln32Ter) in the *IFT27/BBS19* gene in family A. To date, only 8 mutations, including 5 missenses, 2 splice sites, and 1 small deletion, have been reported in the *IFT27/BBS19* gene (HGMD, 2022). The present study reported the first nonsense mutation and the ninth novel mutation (c.94C>T; p.Gln32Ter) in the *IFT27/BBS19* gene. Intraflagellar Transport Protein 27 homolog *IFT27/BBS19* is a member of the RAS oncogene family. *IFT27* encodes intraflagellar transport protein 27, a GTP-binding protein that is a core element of the intraflagellar transport complex B. It is a cytoplasmic protein, mostly expressed in tissues rich with ciliated cells, such as the kidneys and testis. The cytogenetic location of *IFT27* is 22q12.3, with 8 exons and encoding a 186 amino acid protein (NM_006860.5). *IFT27s* function like a small GTPase for the IFT-B complex, regulating the exit of the BBSome from cilia through *ARL6* interaction, preventing aggregation of GTP-free *ARL6*. *ITF27* also interacts with *IFT25* of the IFT-B complex, carrying out its role in intraflagellar transport, flagellar assembly, and maintenance. An animal model zebrafish embryo was co-injected with morpholino oligonucleotides (MO), an expression blocker of *ift27*, and displayed severe renal anomalies and venter body curvature (ciliopathy) due to loss of function and reduction of *ift27* expression [21]. *IFT27* has a vital role in ciliogenesis and the cell cycle (Figure 4). Its partial knockdown shows disruption in the cell cycle (cytokinesis) and cilium biogenesis, while complete knockdown is lethal and will lead to embryonic death. Therefore, partial knockdown of *ift27* was introduced in *Chlamydomonas reinhardtii* by RNA interference (RNAi) and their cilium structure and growing capabilities (cell cycle) were studied. Most of the cells/clones displayed reduced size cilium and were mislocalized. The growth rate was found to be deeply correlated with the amount of *ift27* expression. Approximately 21% failed to grow, 53% showed a reduced level of expression, and about 25% showed a slow growth rate with various division defects compared to the wild type [22]. Moreover, *IFT27* also plays an important role in normal hair follicle formation through the hedgehog (Hh) signaling pathway. These facts suggest that in family A, the present variant (c.94C>T; p.Gln32Ter) might cause the above clinical manifestation, due to loss of function, either through nonsense-mediated mRNA decay (NMD) or due to the production of a truncated protein of *IFT27*.

In family B, WES revealed a novel homozygous nonsense mutation (c.160A>T; p.Lys54Ter) in the *BBIP1/BBS18* gene located on chromosome 10q25.2. To date, only 3 mutations, including 1 missense, 1 nonsense, and 1 splice site, have been reported in the *BBIP1/BBS18* gene (HGMD, 2022). The present study reported the second nonsense mutation and the fourth novel mutation (c.160A>T; p.Lys54Ter) in the *IFT27/BBS19* gene. BBSome interacting protein 1 (*BBIP1*; MIM 613605) is a member of the BBS gene family, having 4 exons, 2057 bp transcript, and encoding for 92 amino acid proteins. It is mostly expressed in the cells of bone marrow, the brain, retina, kidneys, ovaries, and testis tissues. It is mostly localized to the cytoplasm, cytosol, and cilium. *BBIP1* regulate important pathways such as cargo trafficking to the periciliary membrane (cilium biogenesis) and organelle biogenesis/maintenance through the BBSome complex. *BBIP1* is one of the eight core proteins of the BBSome complex, essential for the proper assembly and structural stability of the BBSome complex. A stable BBSome complex acts like a coat complex, sorting out ciliary proteins for ciliogenesis, mediated by the Rab8 GDP/GTP exchange factor. Rab8 GDP/GTP exchange factors are present near the basal body and associated with the BBSome complex [23]. In addition to ciliogenesis, *BBSIP1* also plays a role in Kupffer’s vesicle formation, melanosome transport, acetylation, and stabilization of the cytoplasmic microtubules. Similarly, Scheidecker et al. [24] investigated knockout *bbip1* in zebrafish to study its effects on ciliary morphology. They observed that pronephric cilia were shortened, the pronephros cystic were bilaterally dilated, and Kupffer’s vesicles were abnormal. *BBIP1* expression was not detected in the affected fibroblast cells due to misfolding and malincorporation to the BBSome complex, leading to rapid degradation or NMD. Moreover, they revealed that depletion of the *BBIP1* protein also significantly reduces the incorporation of the *BBS4* protein into the BBSome complex, leading to an aberrant BBSome complex and ciliopathies [24]. 

In family C, a novel homozygous nonsense mutation was observed in the *BBS15/WDPCP* gene with clinical features that overlapped with previous studies such as obesity, mild learning disability, speech difficulties, mild hearing impairment, and rod–cone dystrophy, also reported earlier [25,26]. To date, 61 mutations have been associated with *BBS15/WDPCP* disease pathogenesis, including 16-missense–nonsense mutations, 15 splice sites, 22 small deletions, and 8 small insertions that have been reported in the *BBIP1/BBS18* gene (HGMD, 2022). WD Repeat-Containing and Planar Cell Polarity Effector (WDPCP-BBS15) is a member of the WD repeat Fritz gene family. Its cytogenetic location is 2p15, having 31 exons, 3326 base pairs transcript, and encoding a protein with 713 amino acids. The WD Repeat-Containing and Planar Cell Polarity Effector (WDPCP) with fritz homolog *(Drosophila)* is a cytoplasmic protein, mostly expressed in tissues rich with ciliated cells such as the kidneys, brain, and testes. It is localized mainly to the base of the actin cytoskeleton and to cilia, that is why it controls cell polarity, cell migration, and ciliogenesis [25,26] (Figure 4). WD40 repeat has two domains, a proline-rich domain and a coiled-coil domain. The WD40 repeats a β-propeller structure, which provides a surface for protein–protein interaction and is evolutionarily conserved, while the proline-rich domain is the most divergent part of the WDPCP/Fritz among species. The coiled-coil domain mediates the multimerization of various proteins [27]. 

Family D is a consanguineous family with distinctive hallmark clinical features such as polydactyly, obesity, ID, renal abnormalities, and retinitis pigmentosa; these features overlap with previously reported patients [28,29]. Mesoaxial polydactyly is suggested as the major phenotype in *LZTFL1* (*BBS17*) pathogenesis but was not observed in our patients [28]. Chronic kidney failure reported in the previous two reports was not observed in our patients [28,29]. Using WES, we revealed a novel nonsense mutation (c.505A>T; p.Lys169Ter) in *LZTFL1*. To date, 7 mutations have been associated with *BBS17/LZTFL1,* including 2 missense, 2 nonsense, 1 regulatory mutation, 1 small deletion, and 1 small insertion (HGMD, 2022).

*LZTFL1* encodes a leucine zipper transcription factor-like protein 1, having 10 exons, a 4073 bp transcript, and encoding a 299 amino acid protein (NM_020347.2). The *LZTFL1* is mostly expressed in the cytoplasm and localized ubiquitously. It plays a very vital role in BBSome ciliary trafficking and acts as a negative regulator of sonic hedgehog pathway signaling [30]. LTZFL1 binds to the BBSome in the cytoplasm, inhibits BBSome ciliary entry, and regulates the ciliary trafficking of Smoothened (SMO), a seven-transmembrane hedgehog signal transducer, thus increasing its ciliary localization. The sonic hedgehog pathway (SHH) is a well-known pathway for organizing the body plan and organogenesis [10,31]. The *LTZFL1* gene encodes a ubiquitously expressed protein that localizes to the cytoplasm. This protein interacts with BBS proteins and, through its interaction with BBS protein complexes, regulates protein trafficking to the ciliary membrane (Figure 4). Lztfl1^−/−^ mouse embryonic fibroblasts (MEFs) have significantly longer cilia than wild-type MEFs, and global Lztfl1 deficiency results in pleiotropic phenotypes, including obesity, also observed in the Lztfl1^−/−^ mouse model [32,33].

The *MKKS* gene maps to chromosome 20p12.2 and encodes a protein of 570 amino acids in length (BBS6) [5]. *MKKS* gene variants have been shown to cause Bardet–Biedl syndrome 6 (BBS6; OMIM: 605231) with clinical features, including obesity, polydactyly, pigmentary retinopathy, renal abnormalities, intellectual disability, and hypogonadism with hypertension and diabetes mellitus as secondary characteristics [34] (Figure 4). *MKKS* mutations are also involved in causing McKusick–Kaufman syndrome (MKS; OMIM: 236700), an autosomal recessive disorder [35]. The clinical features of MKS and BBS significantly overlap with each other, with the exclusion of obesity and retinopathy or learning irregularities in MKS syndrome patients [34]. Three domains (equatorial, intermediate, and apical) make up the main body of the *BBS6* protein. The equatorial and apical domains are linked to each other by the intermediate domain. The apical domain functions to bind with the substrate. The three *MKKS*/*BBS6* variants (p.Thr259LeufsTer21; p.Ser40*) identified in families E and I were located in different *MKKS* domains. BBS6 is a chaperonin family member, functions to build the BBSome complex, a hetero-octameric protein complex, and plays a fundamental role in primary cilia homeostasis. It is quite probable that mutant sequence variants in *BBS6* may disrupt the BBSome complex, leading to buckling cilia formation [5]. The *MKKS* variants causing BBS6 have previously been reported in many ethnic populations, including those in China [36], Iran [37], and Pakistan [5,38].

BBS1 is caused by mutations in the *BBS1* gene (MIM: 209901), located on the long arm of chromosome 11 (11q13.2). *BBS1* is expressed in a number of tissues, including fetal, testes, retinal, adipose, cardiac, pancreatic, and skeletal tissues but has its highest rate of expression in the kidneys [39]. *BBS1* variants have a number of effects on different signaling pathways. BBS1 is an essential and critical component of BBSome assembly and plays a fundamental role in ciliary membrane biogenesis by building up the level of Rab8-GTP [40]. Moreover, BBS1 is involved in the retrograde trafficking of ciliary GPCRs in the BBSome complex [41].

We identified a recurrent homozygous missense *BBS1* variant (c.1339G>A; p.Ala447Thr) in two families (F, G). The patients presented the typical features of Bardet–Biedl syndrome, including truncal obesity, polydactyly, intellectual disability, hypogonadism, and retinitis pigmentosa. This missense variant has already been reported in Bangladeshis BBS1 patients [42]. This variant, c.1339 G>A, is located at the terminal nucleotide, a part of the consensus splice site of exon-13 of the *BBS1* gene sequence. Nucleotide substitutions within the consensus splice site regions lead to aberrant splicing [43]. Before this report, missense *BBS1* variant c.1339G>A was never reported in the Pakistani population; this is the first report of this missense variant in the Pakistani population.

In family H, we identified a homozygous donor splice site variant (c.951+1G>A; p?) in the *BBS1* gene with typical phenotypes of Bardet–Biedl syndrome 1. This variant has already been reported in European BBS1 patients [44]. Here, this *BBS1* splice site variant was reported for the first time in the Pakistani population.

In contrast, the identified pathogenic frameshift variants (c.196delA; p.Arg66Glufs*12) in *BBS5* in family J were also reported previously for the Pakistani population. The patients exhibited typical features of BBS reported previously in the literature [6].

In eukaryotes, the cilium is a sensory organelle that performs specialized functions in several cell types. Cilia disruption causes developmental patterning errors, progressive degenerative diseases, and sensory deficiencies in vertebrates. The cilia and flagella are produced and maintained by intraflagellar transport (IFT), a process in which macromolecular protein ‘trains’ consisting of IFT-A and IFT-B subcomplexes traverse the ciliary microtubule axoneme bidirectionally via connection with kinesin and dynein motors. Disease-causing variants in several Bhuman IFT genes have been associated with Bardet–Biedl syndrome (BBS). In addition, all the genes reported in the present study (*IFT27*, *BBIP1*, *WDPCP*, *LZTFL1*, *MKKS*/*BBS5*, *BBS1*) are associated with the kinesin subunit in the cilia. The kinesin subunit plays a vital role in the transportation of different components required for the maintenance and building of flagella and cilia. These vital components are transported from the cell body (site of synthesis) to the distal tip (site of growth) (Figure 4).

BBS is a genetically heterogeneous and highly pleiotropic disorder with very poor genotype-to-phenotype correlations. Clinically and genetically, BBS is a very complex disorder, and proper molecular diagnosis is extremely important to provide an accurate risk assessment and management plan [45]. Different factors involved in incomplete penetrance, variable expression, and different genomic rearrangements that cause BBS are still waiting for new discoveries [46]. Mostly, WES is used in clinical practice to diagnose genetic disorders on a molecular level. Gene-paneling can be an option in such phenotypes; however, WES is cost-effective and common in clinical practice. Similarly, families that lack proper diagnosis as a result of WES can be submitted to whole genome sequencing and other alternative methods to screen for variants [47]. In addition, hotspot mutations can be added to the newborn screening program [48], and for future interventions, techniques such as NIPT, PGT-A, and PGT-M could be adopted for affected families [49,50,51]. Furthermore, building a genetic database of variants [52] will ultimately lead to clinical trials in future [53].

In conclusion, herein, we reported novel and previously reported mutations in 10 families with hallmark features of BBS. The study not only added to the mutation spectrum of BBS, but also helped us to create a database of BBS-associated families and perform proper genetic counselling that might prevent the disease in future. It is possible that these understandings will prevent the pathogenesis of BBS and lead us to design novel strategies for BBS treatment in the near future.

## Figures and Tables

**Table 1:** Clinical evaluation of affected individuals investigated in the present study.

	Family A	Family B	Family C		Family D	Family E		Family F		Family G	Family H		Family I	Family J
Affected individuals	II-1	II-1	II-1	II-3	II-1	II-1	II-3	II-2	II-3	II-1	II-1	II-3	II-2	II-3
Age (years)	12	14	8	11	15	20	17	35	33	32	29	26	16	18
Height (meters)	1.60	1.52	1.44	1.463	1.474	1.40	1.36	1.70	1.60	1.58	1.50	1.45	1.558	1.583
Weight (Kilograms)	78.6	75.8	62	66.3	65.6	65	58	88	83	80	79.75	75	87.6	85.6
BMI (Kg/m^2^)	30.7	32.8	29.2	31	30.2	33.16	31.35	30.44	32.42	32.04	35.44	35.67	36.1	34.2
Major BBS phenotypes														
Retinal degeneration	+	+	+		+	+		+		+	+		+	-
Polydactyly	+	+	+		+	+		+		+	+		+	+
Obesity	+	+	+		+	+		+		+	+		+	+
Developmental delay	+	-	-		+	+		+		+	+		+	+
Hypogonadism	-	+	+		+	+		+		+	+		+	+
Renal abnormality	+	+	+		+	+		+		+	+		-	+
Intellectual disability	-	-	-		-	+		+		+	+		+	+
Minor BBS phenotypes														
Speech disability	+	+	+		+	+		+		+	+		+	+
Strabismus, cataract, astigmatism	+	+	-		+	+		+		-	+		+	-
Brachydactyly	-	-	-		-	-		-		-	-		-	-
Diabetes mellitus	-	-	-		-	+		-		-	-		-	-
Syndactyly	-	+	-		-	-		-		-	-		-	-
Ataxia, imbalance	+	+	+		+	-		-		-	-		-	+
Mild spasticity	-	-	+		-	-		-		-	-		-	-
Heart problems	-	-	-		-	-		-		-	-		-	-
Dental anomaly	-	-	-		-	-		+		-	-		-	-
Liver disorders	-	-	-		-	-		-		-	-		-	-
Hearing Loss	+	-	+		-	-		-		-	-		-	-
Gastro-intestinal complications	-	-	-		-	-		-		-	-		-	-
Dermatologic issues	-	-	-		-	-		-		-	-		-	-

**Table 2:** Pathogenicity scores calculated via in silico tools for different *BBS* variants identified in 10 BBS families.

S. No.	Family ID	Gene	Cytogenic Location	Variant	SIFT	MutationTaster	CADD	VarSome	ACMG 2015
1	A	*IFT27*	22q12.3	c.94C>T; p.Gln32Ter	NA	Disease-causing, Prob: 1	Damaging, Score: 42.0	Pathogenic	Pathogenic (PVS1, PP5, PM2)
2	B	*BBIP1*	10q25.2	c.160A>T; p.Lys54Ter	NA	Disease-causing, Prob: 0.9999	Damaging, Score: 17.68	Uncertain Significance	Uncertain Significance (PVS1, PM2)
3	C	*WDPCP*	2p15	c.720C>A; p.Cys240Ter	NA	Disease-causing, Prob: 0.9963	Damaging, Score: 20.3	Likely Pathogenic	Likely Pathogenic (PVS1, PM2)
4	D	*LZTFL1*	3p21.31	c.505A>T; p.Lys169Ter	NA	Disease-causing, Prob: 0.9967	Damaging, Score: 28.0	Likely Pathogenic	Likely Pathogenic (PVS1, PM2)
5	E	*MKKS*	20p12.2	c.775delA; p.Thr259Leufs*21	NA	Disease-causing, Prob: 1	NA	Pathogenic	Pathogenic (PVS1, PP5, PM2)
6	F	*BBS1*	11q13.2	c.1339G>A; p.Ala447Thr	Benign, Score: 0.556	Disease-causing, Prob: 1	Damaging, Score: 25.0	Pathogenic	Pathogenic (PP5, PS3, PP3, PM2)
7	G	*BBS1*	11q13.2	c.1339G>A; p.Ala447Thr	Benign, Score: 0.556	Disease-causing, Prob: 1	Damaging, Score: 25.0	Pathogenic	Pathogenic (PP5, PS3, PP3, PM2)
8	H	*BBS1*	11q13.2	c.951+1G>A; p?	NA	Disease-causing, Prob: 1	Damaging, Score: 33.0	Pathogenic	Pathogenic (PVS1, PP5, PM2)
9	I	*MKKS*	20p12.2	c.119C>G; p.Ser40*	NA	Disease-causing, Prob: 1	Damaging, Score: 33.0	Pathogenic	Pathogenic (PVS5, PP5, PM2)
10	J	*BBS5*	2q31.1	c.196delA; p.Arg66Glufs*12	NA	Disease-causing, Prob: 1	NA	Likely Pathogenic	Likely Pathogenic (PVS1, PM2)

Abbreviations: NA; Not applicable, Prob; Probability.

## Data Availability

Data are available upon reasonable request. Contact corresponding authors.
